# Inverse Relationship between Progesterone Receptor and Myc in Endometrial Cancer

**DOI:** 10.1371/journal.pone.0148912

**Published:** 2016-02-09

**Authors:** Tamar Kavlashvili, Yichen Jia, Donghai Dai, Xiangbing Meng, Kristina W. Thiel, Kimberly K. Leslie, Shujie Yang

**Affiliations:** 1 Department of Obstetrics and Gynecology, University of Iowa, Iowa City, IA, United States of America; 2 Holden Comprehensive Cancer Center, University of Iowa, Iowa City, IA, United States of America; University of Wisconsin - Madison, UNITED STATES

## Abstract

Endometrial cancer, the most common gynecologic malignancy, is a hormonally-regulated disease. Response to progestin therapy positively correlates with hormone receptor expression, in particular progesterone receptor (PR). However, many advanced tumors lose PR expression. We recently reported that the efficacy of progestin therapy can be significantly enhanced by combining progestin with epigenetic modulators, which we term “molecularly enhanced progestin therapy.” What remained unclear was the mechanism of action and if estrogen receptor α (ERα), the principle inducer of PR, is necessary to restore functional expression of PR via molecularly enhanced progestin therapy. Therefore, we modeled advanced endometrial tumors that have lost both ERα and PR expression by generating ERα-null endometrial cancer cell lines. CRISPR-Cas9 technology was used to delete ERα at the genomic level. Our data demonstrate that treatment with a histone deacetylase inhibitor (HDACi) was sufficient to restore functional PR expression, even in cells devoid of ERα. Our studies also revealed that HDACi treatment results in marked downregulation of the oncogene Myc. We established that PR is a negative transcriptional regulator of Myc in endometrial cancer in the presence or absence of ERα, which is in contrast to studies in breast cancer cells. First, estrogen stimulation augmented PR expression and decreased Myc in endometrial cancer cell lines. Second, progesterone increased PR activity yet blunted Myc mRNA and protein expression. Finally, overexpression of PR by adenoviral transduction in ERα-null endometrial cancer cells significantly decreased expression of Myc and Myc-regulated genes. Analysis of the Cancer Genome Atlas (TCGA) database of endometrial tumors identified an inverse correlation between PR and Myc mRNA levels, with a corresponding inverse correlation between PR and Myc downstream transcriptional targets SRD5A1, CDK2 and CCNB1. Together, these data reveal a previously unanticipated inverse relationship between the tumor suppressor PR and the oncogene Myc in endometrial cancer.

## Introduction

Uterine endometrial cancer is the most common gynecology malignancy. Over 52,000 cases are diagnosed each year, and American women have a lifetime risk of 1:11 [1]. The uterine endometrium is exquisitely sensitive to steroid hormones. Estrogen acting through the estrogen receptor (ER: ERα and ERβ), drives proliferation, while progesterone acts through the progesterone receptor (PR: PRA and PRB) to counteract these effects by inducing differentiation, promoting apoptosis, and inhibiting invasion [2]. Based on the role of progesterone as a tumor suppressor in the endometrium, progestin-based hormonal therapy has been used to treat endometrial hyperplasia and endometrial cancer for more than 60 years [3]. However, hormonal therapy has typically been successful only in tumors where hormone receptors are expressed. Our group and other groups have reported that the expression of PR positively correlates with a favorable prognosis and response to progestin treatment [3,4], whereas loss of PR expression underlies treatment failure. This limits the impact of hormonal therapy in advanced disease where receptors are lost.

In our recently published study, we provide compelling evidence that it is possible to convert a PR-negative cancer into a PR-positive cancer using epigenetic modulators [5]. This approach enhances sensitivity to progestin therapy in tumors with low levels of PR and induces progestin sensitivity in tumors that were never responsive. We propose that the efficacy of progestin therapy can be significantly enhanced by combining it with epigenetic modulators, which we term “molecularly enhanced progestin therapy.”

To investigate if this molecularly enhanced progestin therapy can be applied to advanced endometrial cancer patients with ERα and PR negative tumors; we generated ERα null endometrial cancer cell lines by applying novel CRISPR-Cas9 technology to delete ERα at the genomic level (ESR1). Our data demonstrate that treatment with an HDACi is sufficient to restore functional PR expression, even in cells devoid of ERα.

Our studies also revealed a previously unanticipated inverse correlation between PR and the oncogene Myc in endometrial cancer, suggesting that HDACi treatment provides an additional advantage of Myc downregulation. We report an in-depth analysis of this correlation and hypothesize that loss of myc underlies the differentiating effects of progestin therapy.

## Materials and Methods

### Antibodies and Reagents

Estradiol (#E2257) and progesterone (#P6149) were obtained from Sigma Aldrich and resuspended in ethanol. Panobinostat (LBH589) was purchased from Selleck Chemicals and resuspended in DMSO. Antibodies against PRA/B (#3153), PRB (#3157), FOXO1 (#2880) and Myc (#13987) were from Cell Signaling. HSP90 antibody (ADI-SPA-835) was from Enzo. ERα antibody (sc-8002) was from Santa Cruz. β-actin antibody (#A1978) was obtained from Sigma Aldrich.

### Cell Lines and Cell Culture

ECC-1 endometrial cancer cells were purchased from ATCC and grown according to the recommended guidelines. Ishikawa H endometrial cancer cells [6] (gift from Dr. Erlio Gurpide, New York University) were grown in DMEM media supplemented with 10% fetal bovine serum (regular FBS, r-FBS) or charcoal-stripped FBS (cs-FBS, life technologies, #12676–011) and penicillin-streptomycin (Gibco). Before addition of progesterone (P4) to cells, cell growth media with 10% FBS was replaced with growth media containing 5% charcoal-stripped serum to remove steroids from FBS.

### Generation of ERα Knockout Cells

Genomic deletions of ERα (ESR1) were created in ECC1 cells using three pairs of chimeric single guide RNAs (sgRNAs) as previously described [7]. Briefly, sgRNA oligos (S1 Table) were synthesized and cloned into the lentiCRISPR transfer plasmid for virus production. Viral vectors were produced in HEK293T cells, followed by infection of ECC1 cells as described [7]. ECC1-ESR1 CRISPR knockout (KO) cells were selected with 2 μg/ml puromycin after 48 hours of transduction. ECC1-ESR1 CRISPR KO cells were grown at low density for 2 weeks to select clones. ERα expression was determined by Western blotting to identify clones with no or low ERα protein expression. ECC1-ESR1 CRISPR KO clones were maintained in media supplemented with 5 μg/ml puromycin.

### Adenoviral Expression of PR

ECC1-ESR1 CRISPR KO clone 1–12 and 2–12 was infected with adenoviral vectors encoding PRA, PRB, or PRA/B or an empty vector control (Adcontrol) using a multiplicity of infection (MOI) of 10 viral particles per cell as previously described [8]. An MOI of 10 viral particles per cell was employed to obtain PR expression levels roughly equivalent to the late proliferative phase of the menstrual cycle.

### Real-Time PCR

Total RNA was extracted from cultured cells using the miRvana miRNA Isolation kit (Ambion, Life Technologies). RNA yield and purity was assessed using a NanoDrop Model 1000 spectrophotometer (Thermo Scientific). Total RNA (500 ng) was oligo-dT reverse transcribed with SuperScript III (Invitrogen, Life Technologies). Real-time PCR was performed in triplicate on an Applied Biosystems Model 7900 Genetic Analyzer under standard conditions. Primer sequences are listed in S1 Table. Results were quantitated using the comparative cycle threshold (ΔΔCt) method [9]. Data were normalized to 18S rRNA.

### Western Blotting

Following treatment, cells were solubilized in cold NP-40 cell lysis buffer (150 mM NaCl, 50 mM Tris/HCl, pH 7.4, 1% NP-40 with a protease and phosphatase inhibitor cocktail from Pierce) and then sonicated to release nuclear proteins. Lysates were analyzed by Western blotting with specific primary and HRP-conjugated secondary antibodies. For detection of PR protein, a combination of the PRA/B and PRB antibodies at a 1:1000 dilution each was used.

### TCGA Data Analysis

Patient information was downloaded from The Cancer Genome Atlas Data Portal (https://tcga-data.nci.nih.gov/tcga/) maintained by National Cancer Institute and National Human Genome Research Institute. Gene expression was assayed based on RNASeq conducted on the Illumina platform and was downloaded from NCI’s Cancer Genomics Hub (https://cghub.ucsc.edu/). The calculated expression was for all reads aligning to a particular gene per sample. There were a total of 379 endometrial cancer patients eligible for gene expression analysis. Patients were divided into four groups: endometrioid endometrial carcinoma grade 1, grade 2, grade 3 and serous grade 3 which includes cases designated as high grade and mixed histology type.

### Statistical Analysis

Student’s t-test was used for comparisons of two groups. For TCGA data analysis, statistical analysis and data plotting were conducted with R Studio, and correlation analysis was performed with Spearman method. Statistical significance was concluded with p value < 0.05.

## Results

### Modeling Loss of ERα in Endometrial Cancer Cells

**Fig 1:**
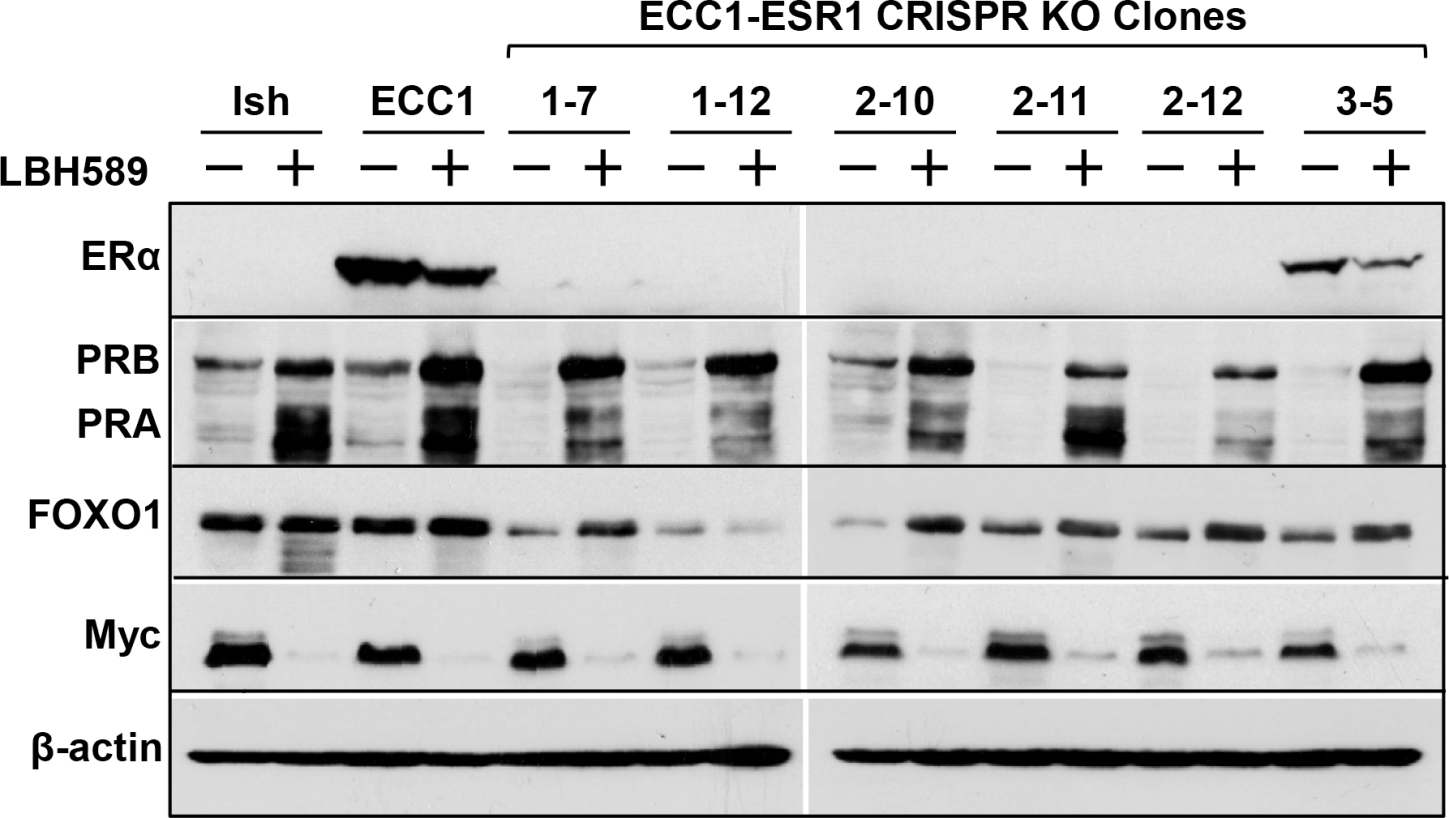
HDAC inhibitor (panobinostat, LBH589) upregulates PR expression and downregulates Myc in ERα knockout endometrial cancer cells lines. Using CRISPR-Cas9 technology, ERα was silenced at the genomic level in ECC1 cells. Ishikawa, parental ECC1 cells and individual ESR1 KO ECC1 clones were treated with 20 nM LBH589 for 24 hr. Expression of ERα, PR, FOXO1, and Myc were evaluated by Western blotting. β-actin serves as a loading control.

To mimic more advanced endometrial cancer which is associated with loss of ERα and PR, we applied CRISPR-Cas9 technology to delete ERα at the genomic level (ESR1) in the ERα- and PR-positive ECC1 endometrial cancer cells using three different sgRNA sequences [7]. These cells are denoted as ECC1-ESR1 CRISPR knockout (KO) cells. We next selected individual clones from cells transfected with each of the three sgRNAs. We confirmed that the majority of clones had complete deletion of ERα at the protein level, though clone 3–5 had a low level of residual ERα as compared to parental ECC1 cells (Fig 1). Consistent with ERα as the principle inducer of PR expression, loss of ERα resulted in loss of PR as well as a reduction in the PR target gene FOXO1 (Fig 1).

### HDAC Inhibitor Restores PR Expression in ECC1-ESR1 CRISPR KO Endometrial Cancer Cells Lines

We next asked if ERα expression is required for HDACi-mediated induction of PR, which we previously reported as a mechanism to restore sensitivity to progestin-based therapy [5,10]. In all ECC1-ESR1 CRISPR KO clones, treatment with the pan-HDAC inhibitor LBH589 (panobinostat, Novartis) substantially increased PR protein expression and activity as determined by expression of the PR target gene, FOXO1 (Fig 1). Note that cells were maintained in serum that contains progesterone. As a control, we also examined PR levels in parental ECC1 cells and Ishikawa cells, which have modest baseline PR expression. Consistent with our previous studies [5,10], LBH589 promoted robust PR expression in these cell lines. Taken with our previously published data that the combination of LBH589 and progesterone decreases colony formation by parental Ishikawa cells [5], these data suggest that, even in patients with loss of both ERα and PR, it may be possible to achieve sensitivity to progestin therapy with an epigenetic modulator.

### HDAC Inhibitor Represses Oncogene Myc Expression in Endometrial Cancer Cells

As a control, we also assessed expression of the oncogene Myc based on several reports that ERα and PR promote transcription of Myc in breast cancer [11–13]. Unexpectedly, expression of Myc was unchanged in untreated ECC1-ESR1 CRISPR KO cells despite loss of ERα and PR expression. However, HDACi treatment, in addition to inducing PR as described above, completely repressed Myc protein expression. We also observed a decrease in Myc and an upregulation of PR in the parental ECC1 cells as well as another endometrial cancer cell line, Ishikawa, following treatment with LBH589. These data identify an inverse relationship between Myc and PR in endometrial cancer cells.

**Fig 2:**
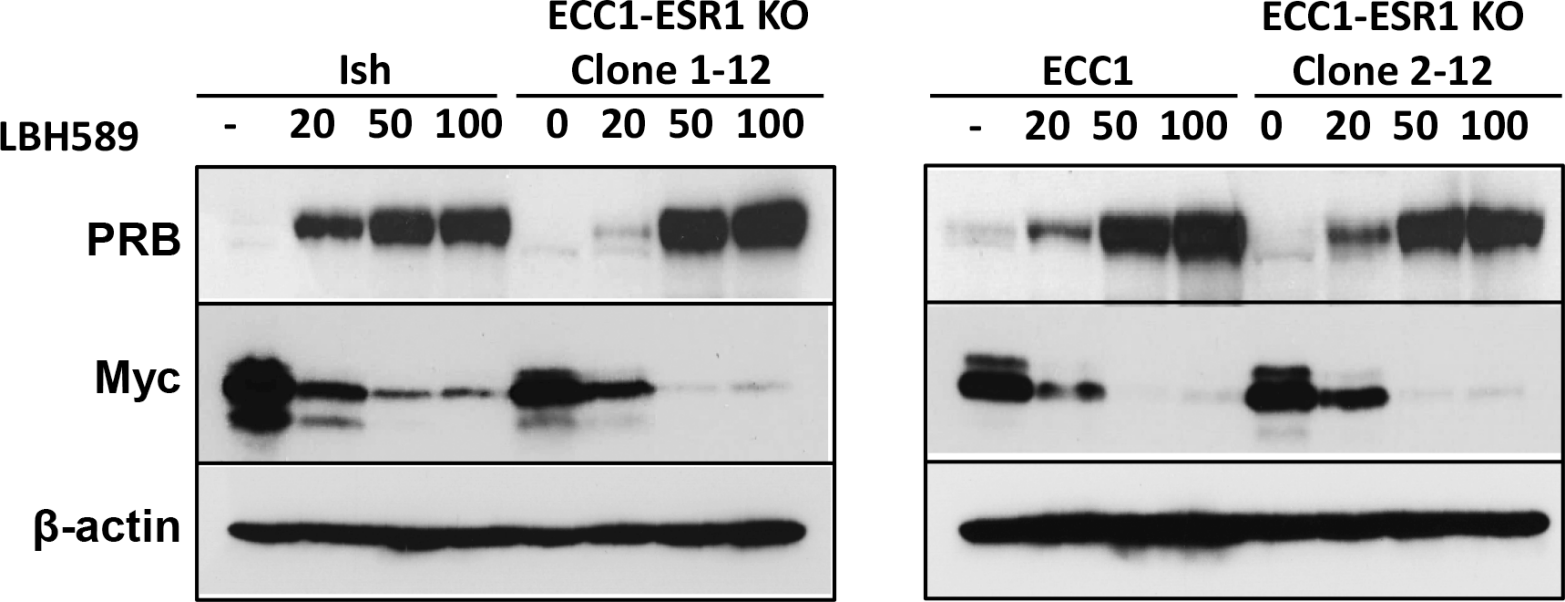
Higher concentrations of HDACi LBH589 further increase PR and decrease Myc expression in endometrial cancer cells. Cells were treated with indicated concentrations of LBH589 for 24 hr and protein expression analyzed by Western blotting. β-actin serves as a loading control.

We also utilized higher concentrations of LBH589 to determine if Myc levels could be further suppressed. In both parental and ECC1-*ESR1* CRISPR KO endometrial cancer cells, PR levels were progressively elevated in response to increasing concentrations of LBH589, with a corresponding dose-dependent decrease in Myc expression (Fig 2). These findings confirm our observation of the inverse correlation between PR and Myc expression in Fig 1 and suggest that higher concentrations of LBH589 may be required to restore PR levels in tumors with long-term loss of hormone receptor expression.

### Estrogen Stimulation Increases PR and Decreases Myc Expression in Endometrial Cancer Cell Lines

**Fig 3:**
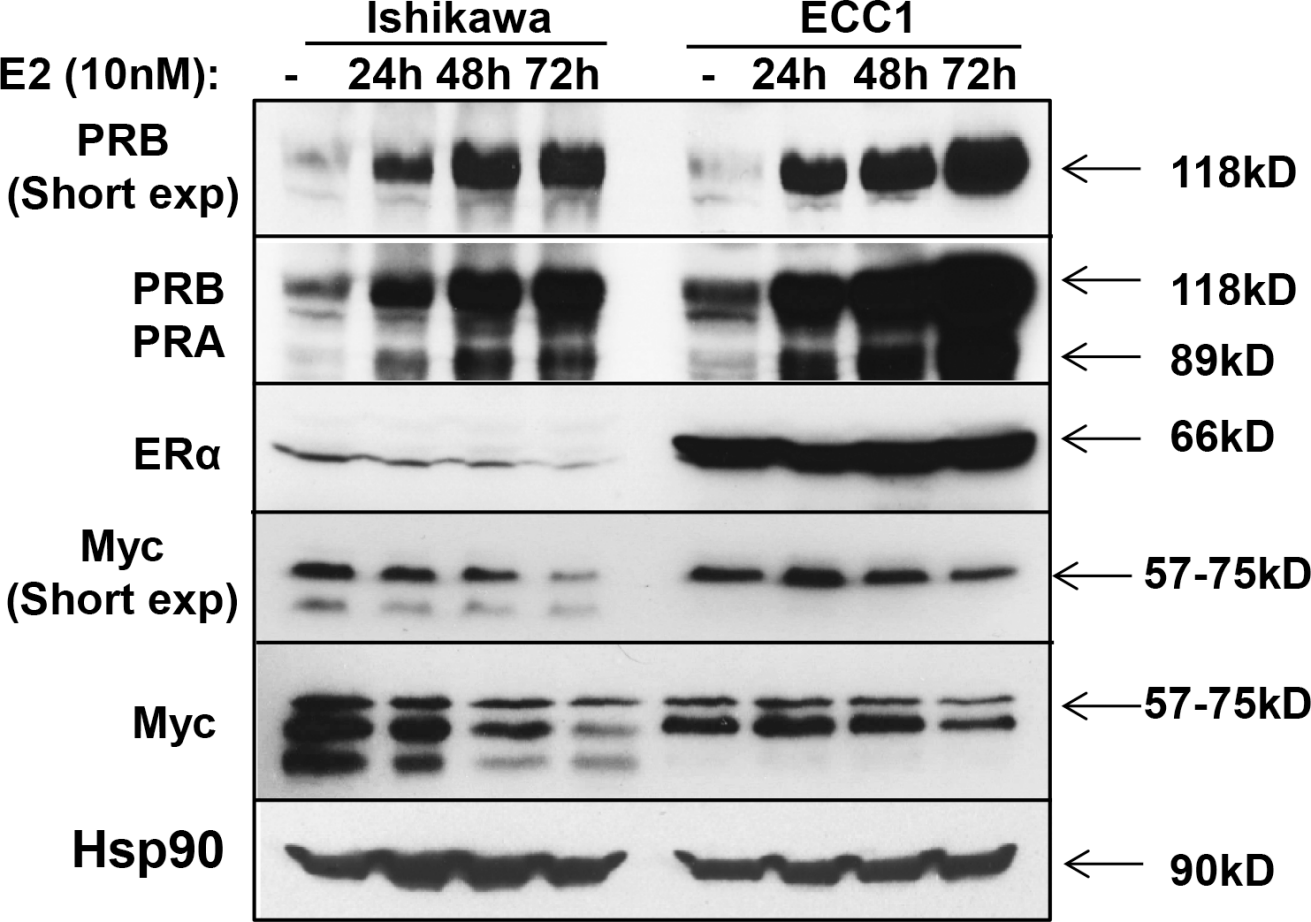
Activation of ERα with estrogen simultaneously increases PR expression and decreases Myc expression in endometrial cancer cells. Ishikawa and ECC1 cells were treated with 10 nM estradiol (E2) for indicated times. PR, Myc and ERα protein expression was measured by Western blotting. Hsp90 serves as loading control.

The inverse correlation between PR and Myc expression in Figs 1 and 2 challenges the well-accepted paradigm in breast cancer that PR promotes Myc expression [12,14]. We first confirmed that estrogen stimulation of MCF7 cells, which contain endogenous ERα and PR, results in robust expression of PR and Myc, with anticipated ligand-mediated ERα downregulation (S1 Fig). Addition of progesterone further increased Myc levels, in line with previous studies [12,14]. By contrast, estrogen stimulation of endometrial cancer cells with endogenous ERα expression induced PR expression, with a concomitant decrease (Ishikawa cells) or no further increase (ECC1 cells) in Myc protein levels (Fig 3).

### PR Negatively Regulates Myc Expression and Activity in Endometrial Cancer Cell

**Fig 4:**
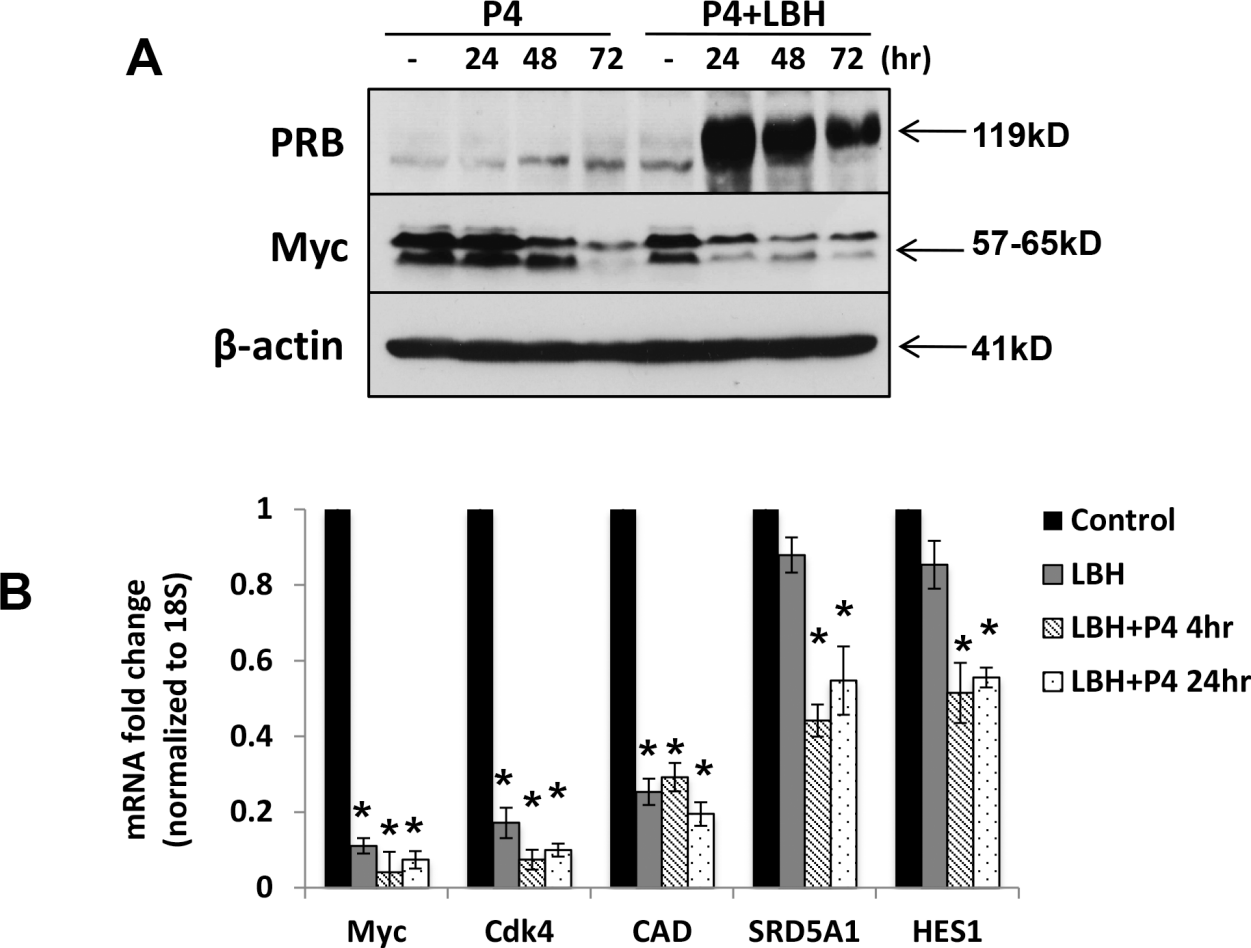
The combination of HDACi and progesterone negatively regulates Myc expression and activity in endometrial cancer cells. (A) Ishikawa cells were treated with 20 nM LBH589, 100 nM progesterone (P4) or the combination for the indicated times. PR and Myc protein expression was measured by Western blotting. β-actin serves as a loading control. (B) Expression of Myc and its downstream genes (CDK4, CAD, SRD5A1 and HES1) were analyzed by qRT-PCR in Ishikawa cells were treated with 20 nM LBH589 -/+100 nM P4 for 4 or 24 hrs. Data were normalized to 18S and calculated as the fold change relative to control. * p < 0.05 vs. control.

To investigate if PR mediates Myc downregulation in endometrial cancer cells, we treated Ishikawa cells, which have low baseline ERα and PR levels (Fig 3), with LBH589 in the absence or presence of progesterone for 0–72 hrs. Experiments were performed in charcoal-stripped serum to remove hormones. Treatment with progesterone produced a time-dependent decrease in Myc protein levels, and the addition of HDACi accentuated this effect (Fig 4A). We also assessed Myc transcriptional activity by examining mRNA levels of Myc target genes. Consistent with Western blot data, mRNA levels of Myc were decreased by more than 90% following treatment with LBH589, and addition of progesterone resulted in a further decrease (Fig 4B). The Myc target genes CDK4, CAD, SRD5A1 and HES1 were repressed in response to LBH589+progesterone (Fig 4B).

**Fig 5:**
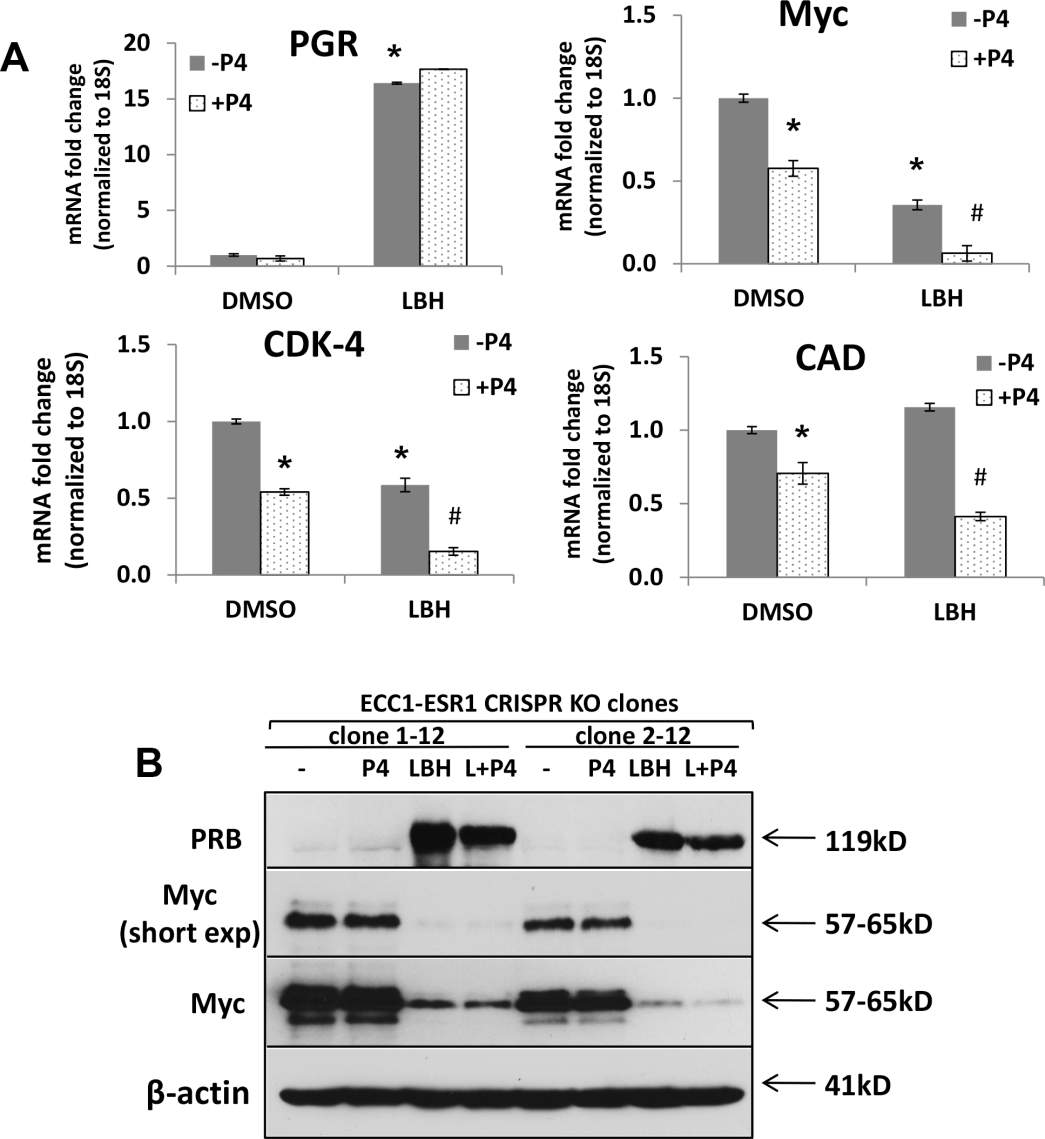
Downregulation of Myc and downstream target genes in response to HDACi alone and in combination with progesterone. (A) ECC1-*ESR1* CRISPR KO clone 1–12 was treated with 100 nM P4 for 16 hr, 20 nM LBH589 for 24 hr, or the combination, followed by analysis of PR, Myc, and PR target genes by qRT-PCR. Data were normalized to 18S and calculated as the fold change relative to control. Results are representative of 3 independent experiments; * p < 0.05 vs. DMSO; # p < 0.05 vs. LBH589. (B) ECC1-*ESR1* CRISPR KO clones were treated as in (A), followed by analysis of PR and Myc protein levels by Western blotting. β-actin serves as a loading control.

To investigate if PR mediates Myc downregulation in ERα-null endometrial cancer cells, molecularly enhanced progestin therapy was applied in ECC1-*ESR1* CRISPR KO cells. Consistent with Fig 4, similar results were obtained in ERα-null cancer cells (Fig 5). Specifically, treatment with LBH589 increased expression of PR and decreased expression of Myc and its downstream transcriptional targets, CDK4 and CAD. This effect was amplified by addition of progesterone (Fig 5A). This loss of Myc expression was confirmed at the protein level (Fig 5B).

**Fig 6:**
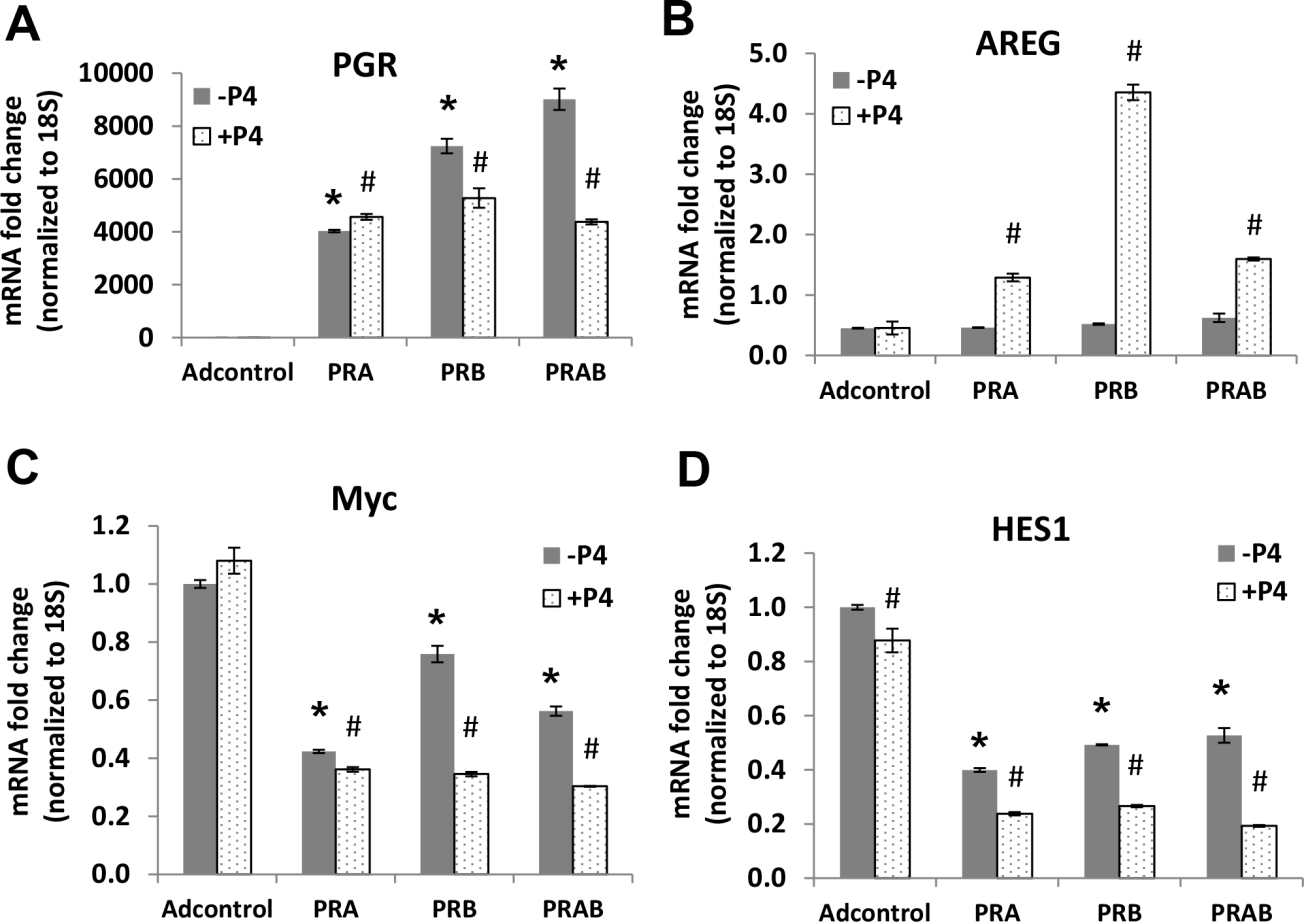
Overexpression of PR decreases Myc expression and activity. ECC1-*ESR1* CRISPR KO clone 1–12 was transduced with control adenovirus (Adcontrol) or adenovirus containing PRA, PRB, or PRA+PRB for 24 hr, followed by 100 nM P4 for an additional 24 hr. mRNA levels were quantified by qRT-PCR, normalized to 18S, and data expressed as fold change relative to Adcontrol in the absence of P4. Results are representative of 3 independent experiments; * p < 0.05 vs. Adcontrol; # p < 0.05 vs.–P4 in the same group.

To provide further evidence for the link between PR and Myc downregulation, we overexpressed PRA and PRB in ECC1-*ESR1* CRISPR KO clone 1–12. We first confirmed that exogenous expression of PRA or PRB by adenoviral transduction leads to robust overexpression of PR and also induction of PR downstream gene AREG (Fig 6). Overexpression of PR resulted in Myc downregulation, and addition of progesterone further decreased mRNA levels of Myc and the Myc target gene, HES1 (Fig 6). These data support our hypothesis that PR negatively regulates Myc expression in endometrial cancer.

### Inverse Correlation between PR and Myc in Endometrial Patient Tumors

**Fig 7:**
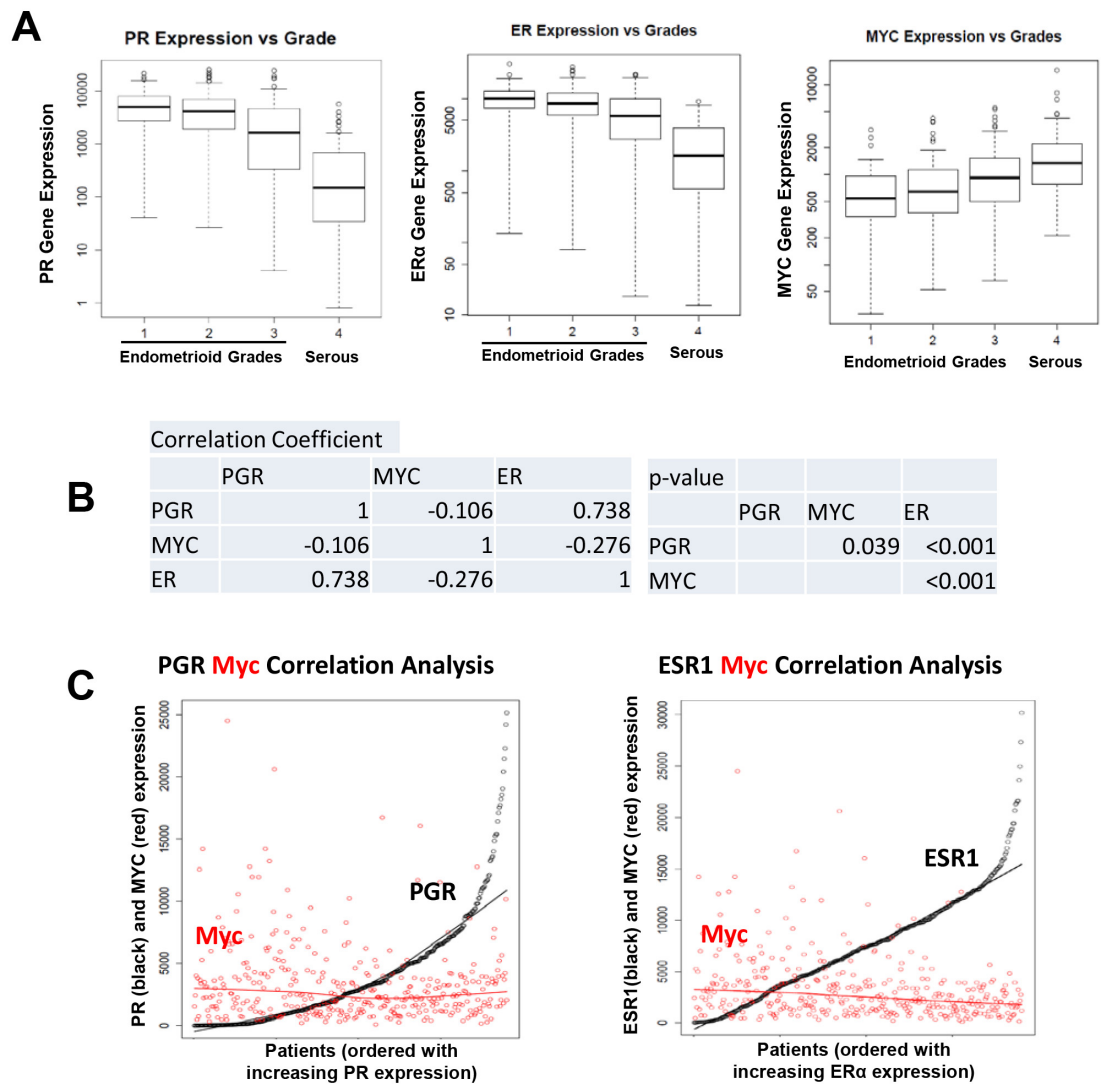
Inverse correlation of Myc with ESR1 and PGR in endometrial cancer TCGA data. (A) Boxplot of mRNA expression of PGR (left panel), ESR1 (middle panel) and Myc (right panel) was analyzed according to endometrial cancer grade in 379 endometrial cancer patient tumors from TCGA database. Patients were divided into four groups: endometrioid type I grade 1 (G1, n = 86), grade 2 (G2, n = 103), grade 3 (G3, n = 123) and serous type II grade 3 (G3, n = 67). (B) Pairwise correlation analysis of PGR, ESR1 and Myc mRNA levels using Spearman method. The correlation coefficients and p values are shown. (C) Scatterplot to show the negative correlation in expression of two genes. Patients were plotted on the x-axis in order of increasing expression of a specific gene. Two regression lines were produced by simple linear regression analysis based on the expression of respective genes. Left: Expression of Myc (red) against PGR (black); right: expression of Myc (red) against ESR1 (black).

To investigate if there is an inverse correlation between PR and Myc expression in endometrial cancer patient samples, we analyzed RNA-Seq data from 379 endometrial tumors in the TCGA dataset. We observed a progressive decrease in PGR and ESR1 mRNA expression from endometrioid endometrial cancers to more aggressive serous tumors as defined by grade level (Fig 7A). On the contrary, Myc mRNA expression was increased significantly in high grade tumors relative to lower grades. The Spearman correlation method confirmed that the correlations of PGR with Myc and ESR1 with Myc are significant (Fig 7B and 7C).

**Fig 8:**
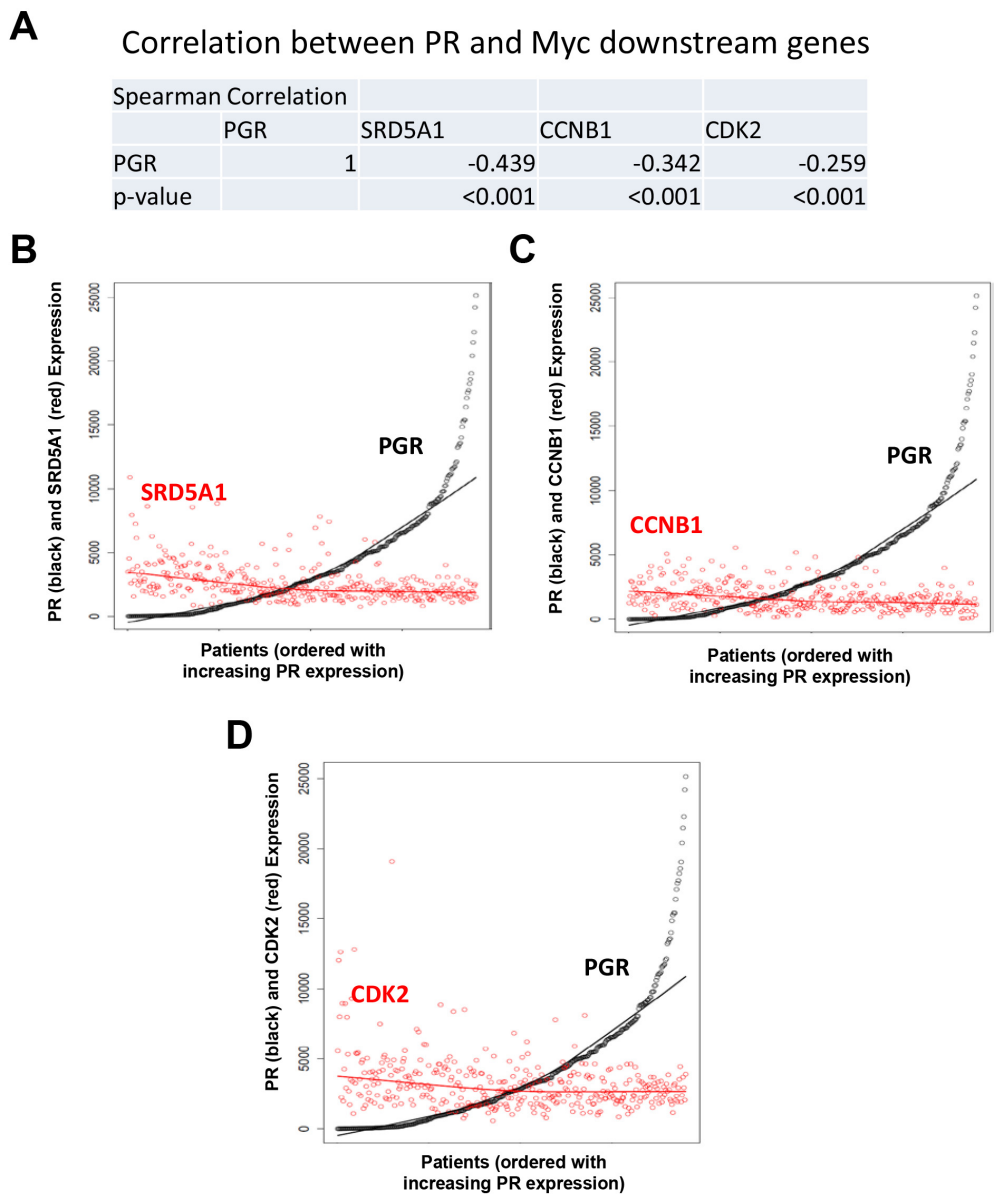
Inverse correlation of PR with Myc target genes in endometrial cancer TCGA data. (A) Pairwise correlation analysis of PGR and Myc downstream genes, SRD5A1, CCNB1 and CDK2 using Spearman method. The correlation coefficients and p values are shown. (B-D) Scatterplots to show the positive or negative correlation of expression of two genes as in Fig 6C. (B) Expression of SRD5A1 (red) against PGR (black); (C) expression of CCNB1 (red) against PGR (black); and (D) expression of CDK2 (red) against PGR (black).

To further validate the inverse correlation between PR and Myc in patient samples, we examined how PR correlated with Myc downstream genes SRD5A1, CCNB1 and CDK2. The Spearman correlation method demonstrated a strong negative correlation of PGR with SRD5A1, CCNB1 and CDK2 expression (Fig 8).

## Discussion

Endometrial cancer is a hormone regulated cancer wherein estrogen drives growth and progesterone suppresses proliferation and leads to differentiation. Response to progestin-based therapy positively correlates with hormone receptor expression, in particular progesterone receptor (PR), yet many advanced tumors are devoid of PR expression as well as ERα, the primary transcriptional inducer of PR. We recently found that PR expression can be restored by HDACi. Herein we extend these findings by reporting that PR expression can be restored in endometrial cancer cells, even those without ERα. These data are critical because they suggest that, even in patients with loss of both ERα and PR, it may be possible to restore sensitivity to progestin through combination treatment with an epigenetic modulator. Surprisingly, we also found that the oncogene Myc, which is known to be induced by ERα and PR in breast cancer cells, was markedly repressed by HDACi treatment, including in cells that lack ERα. Moreover, exogenous expression of PR by adenovirus promoted Myc downregulation, providing further evidence that PR negatively regulates Myc expression. Consistent with the observations in cancer cells, we found that PR and Myc are inversely correlated in endometrial tumors in TCGA dataset. We hypothesize that loss of Myc underlies the differentiating effects of progestin therapy in endometrial cancer.

Myc is a well-characterized oncogene that plays a major role in the pathogenesis of many cancers [15,16]. Like other cancer types, Myc is highly expressed in endometrial tumors [17]. Studies in transgenic mouse models reveal that Myc inactivation results in rapid tumor regression and is frequently associated with hallmarks of cellular differentiation and apoptosis [18,19]. Despite clear evidence for the highly oncogenic role of Myc in many tumor types, including endometrial cancer, a specific small molecule inhibitor of Myc has remained elusive [20]. Current drug development strategies include targeting pathways upstream or downstream of Myc, such as the bromodomain and extra-terminal motif (BET) inhibitor. Indeed, inhibition of BET in multiple myeloma results in remarkable downregulation of Myc expression and associated cell death [16]. Herein we identify another potential mechanism to blunt the oncogenic effects of Myc: PR-mediated suppression of Myc transcription. Therefore, molecularly enhanced progestin therapy not only enhances sensitivity to progestin by restoring functional PR expression [5,10], but also provides an additional advantage of downregulating Myc.

Others have shown that Myc expression is regulated through epigenetic mechanisms. For example, HDACi-mediated repression of Myc expression has also been reported in head and neck squamous cell carcinoma cell lines [21]. The potential mechanism is up-regulation of *MXI1* and *SSB2*, which inhibit the transcriptional activity of Myc [21]. However, our data suggest that the mechanism of Myc repression is through PR. First, treatment with progesterone in combination with HDACi produced a further decrease in Myc as compared to HDACi alone. Second, overexpression of PRA/B by adenovirus in ECC1-*ESR1* CRISPR KO cells substantially decreased Myc expression in the absence of an HDACi, indicative of a PR-mediated effect rather than a general epigenetic effect.

Several reports in breast cancer demonstrate that ERα and PR promote transcription of Myc through binding the estrogen response element (ERE) and progesterone response element (PRE) in the Myc promoter region [11,13,22,23]. The earliest report of a PRE in the Myc promoter region in T47D breast cancer cells was published in 1997 [14]. The observation that ERα and PR induce Myc expression is consistent with the phenomenon that progestins used in contraception drugs promote breast cancer growth *in vitro* and *in vivo* [24–26]. Here we report the converse effect in endometrial cancer, where expression of Myc inversely correlated with ERα and PR, both in endometrial cancer cell lines and in tumors from TCGA database. Control experiments in MCF7 breast cancer cells confirmed the positive correlation of Myc with ERα and PR in this cell type. Thus, the negative regulation of Myc by PR in endometrial cancer may provide one explanation for the opposing effects of progesterone on proliferation in breast vs. endometrial cancer.

In summary, we found that HDACi treatment of endometrial cancer cells provides the dual advantages of upregulating the tumor suppressor PR and downregulating the oncogene Myc. Unfortunately, single agent HDACi has limited clinical efficacy in solid tumors, and emerging studies are exploring the use of HDACi to “prime” tumors to other treatments [27, 28]. Based on our extensive studies of mechanisms of PR silencing in endometrial cancer, we suggest that HDACi can be used to prime endometrial tumors for responsiveness to progestin-based therapy, which we refer to as molecularly enhanced progestin therapy, via epigenetic modulation. Future studies are necessary to understand if combining an HDACi with progestin will restore PR, downregulate Myc, and lead to disease management. A first step may be to explore this combinatorial strategy in the neoadjuvant setting, which would provide the opportunity to examine PR and Myc levels in pre-treatment biopsies and in post-treatment surgical specimens obtained at the time of tumor cytoreduction. Another therapeutic opportunity is in the setting of advanced or recurrent endometrial cancer, where the majority of endometrial tumors have lost expression of PR. By integrating the epigenetic therapies into hormonal regimens, it may be possible to improve outcomes for endometrial cancer, a disease that is on the rise.

## Supporting Information

S1 Fig E2 stimulation induce PR and Myc expression in MCF7 breast cancer cells. MCF7 cells were grown in charcoal stripped serum and treated with 10nM Estroadiol (E2) as indicated time point. PR and ERα protein expression was measured by Western blotting. β-actin serves as loading control.
(TIF)

S1 Table ESR1 sgRNA sequence and primers for real-time PCR. (DOCX)
