# Supplementary figures and images for: Inverse Relationship between Progesterone Receptor and Myc in Endometrial Cancer
Source: PLoS One. 2016 Feb 9;11(2):e0148912. doi: 10.1371/journal.pone.0148912 (PMC4747472; doi:10.1371/journal.pone.0148912)

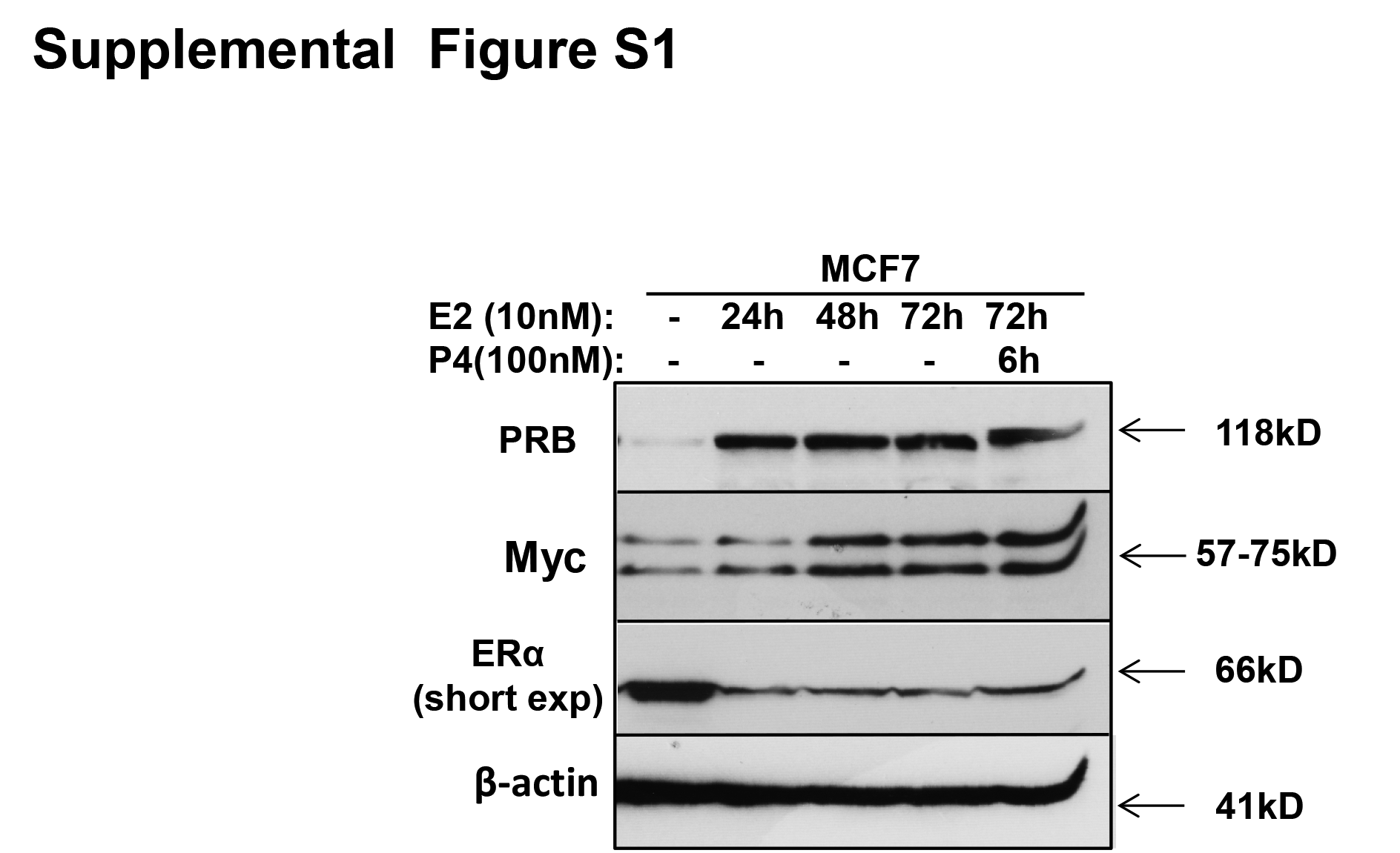

Supplement: S1 Fig — MCF7 cells were grown in charcoal stripped serum and treated with 10nM Estroadiol (E2) as indicated time point. PR and ERα protein expression was measured by Western blotting. β-actin serves as loading control. (TIF) [file pone.0148912.s001.tif]
