# Supplementary material for: Inverse Relationship between Progesterone Receptor and Myc in Endometrial Cancer
Source: PLoS One. 2016 Feb 9;11(2):e0148912. doi: 10.1371/journal.pone.0148912 (PMC4747472; doi:10.1371/journal.pone.0148912)
Supplement: S1 Table — (DOCX) [file pone.0148912.s002.docx]

**S1 Table: ESR1 sgRNA sequences and primers for real-time PCR.**

|  | **sgRNA sequence** | |
| --- | --- | --- |
| ESR1 sgRNA oligo1 | AAATTCAGATAATCGACGCC | |
| ESR1 sgRNA oligo2 | GTGTGCAATGACTATGCTTC | |
| ESR1 sgRNA oligo3 | CTCCGTAAATGCTACGAAGT | |
| **Primer name** | **Forward primer** | **Reverse primer** |
| PGR | ATGTGGCAGATCCCACAGGAGTTT | ACTGGGTTTGACTTCGTAGCCCTT |
| AREG | GTGGTGCTGTCGCTCTTGATA | CCCCAGAAAATGGTTCACGCT |
| PAEP | GAGATCGTTCTGCACAGATGG | CGTTCGCCACCGTATAGTTGAT |
| 18S | AACTTTCGATGGTAGTCGCCG | CCTTGGATGTGGTAGCCGTTT |
| Myc | GGCTCCTGGCAAAAGGTCA | CTGCGTAGTTGTGCTGATGT |
| CDK4 | TCAGCACAGTTCGTGAGGTG | GTCCATCAGCCGGACAACAT |
| CAD | AGTGGTGTTTCAAACCGGCAT | CAGAGGATAGGTGAGCACTAAGA |
| SRD5A1 | CATTGTGCAGTGTATGCTGATGA | GCCACACCACTCCATGATTTC |
| HES1 | CGGACATTCTGGAAATGACA | GTGCGCACCTCGGTATTAAC |
